# Did Lizards Follow Unique Pathways in Sex Chromosome Evolution?

**DOI:** 10.3390/genes9050239

**Published:** 2018-05-03

**Authors:** Shayer Mahmood Ibney Alam, Stephen D. Sarre, Dianne Gleeson, Arthur Georges, Tariq Ezaz

**Affiliations:** Institute for Applied Ecology, University of Canberra, Canberra 2616, Australia; stephen.sarre@canberra.edu.au (S.D.S.); dianne.gleeson@canberra.edu.au (D.G.); georges@aerg.canberra.edu.au (A.G.)

**Keywords:** lizards, genotypic sex determination (GSD), sex-chromosome evolution

## Abstract

Reptiles show remarkable diversity in modes of reproduction and sex determination, including high variation in the morphology of sex chromosomes, ranging from homomorphic to highly heteromorphic. Additionally, the co-existence of genotypic sex determination (GSD) and temperature-dependent sex determination (TSD) within and among sister clades makes this group an attractive model to study and understand the evolution of sex chromosomes. This is particularly so with Lizards (Order Squamata) which, among reptiles, show extraordinary morphological diversity. They also show no particular pattern of sex chromosome degeneration of the kind observed in mammals, birds and or even in snakes. We therefore speculate that sex determination sensu sex chromosome evolution is labile and rapid and largely follows independent trajectories within lizards. Here, we review the current knowledge on the evolution of sex chromosomes in lizards and discuss how sex chromosome evolution within that group differs from other amniote taxa, facilitating unique evolutionary pathways.

## 1. Introduction 

Sex chromosomes, modes of sex determination and reproduction in reptiles (non-avian: tuatara, lizards, snakes, turtles and crocodilians) are among the most diverse of the amniotic vertebrates (reptiles, birds and mammals), often showing little regard to phylogeny, which in turn implies multiple and independent origins as well as frequent transitions and reversals [1,2,3,4,5,6,7]. For example, reptile sex chromosomes vary greatly in their level of degeneration, ranging from cryptic to highly differentiated [6,8,9,10,11] and are the only amniotes where genotypic sex determination (GSD) and temperature-dependent sex determination (TSD) appear to have evolved independently many times with evidence of both modes at the level of order through to species and even within a single species [12,13,14,15,16,17]. Moreover, in some species, like the Australian bearded dragon lizard *Pogona vitticeps* [17,18,19], genes on the sex chromosomes interact with the incubation environment to determine sex. Reptiles also epitomize the variability of modes of reproduction and fertilization among amniotes, with many species exhibiting oviparity, ovoviviparity or placental viviparity, and both facultative and obligate parthenogenesis have been reported [4,20,21,22,23,24,25,26,27,28]. Apart from fishes, no other vertebrate group shows such diversity and variability in the mode and mechanism of sex determination or in sex chromosomes and modes of reproduction. This diversity, all within a single taxonomic order, provides a fertile field for the discovery of novel mechanisms that define the most fundamental of phenotypes, sex. It is therefore remarkable that little is known of the molecular mechanisms that have enabled this diversity. In comparison to other amniote groups, little effort has been made to better understand the mode and mechanisms of sex determination in reptiles, and how they have evolved over 300 million years since they diverged from other amniotes [29,30]. 

In this review, we present a brief overview of the current understanding of sex chromosomes and sex determination in reptiles, particularly in lizards and highlight the aspects that are unique to them. Specifically, we ask: did reptile sex chromosomes follow the same evolutionary pathway as proposed for birds and mammals? 

## 2. Sex Chromosome Evolution in Amniotes—The Classical Concept

Sex chromosomes are the most dynamic entity within a genome, being characterized by a unique morphology and specialized evolution [1,2,3,7,31]. The morphological diversity of amniotic sex chromosomes is truly remarkable ranging from cryptic to highly heteromorphic. Amniote GSD species are either male heterogametic where males and females have XY/XX sex chromosomes (respectively—as in most mammals), or female heterogametic (male/female: ZZ/ZW) as in all birds. Among mammals, monotremes (platypus and echidnas) present as rare exceptions, possessing a remarkable multiple XY system that is not homologous with therian XY systems, but rather show homology with chicken Z chromosomes [32,33]. In contrast to the conservative nature of mammals and bird sex chromosomes, many reptiles exhibit remarkable variation in the sex chromosome pair, and in the system of heterogamety, sometimes even among closely related species or even within populations [6,12,31]. 

It is generally accepted that heteromorphic sex chromosomes originate from an autosomal ancestor following mutational acquisition of a sex determining allele [2,31,34,35,36,37,38]. This can happen within all systems that already involve sex chromosomes or sex determination genes or within those that require environmental factors such as temperature to resolve sex. Additional sex-linked mutations and the subsequent suppression of recombination (either by chromosomal rearrangements or accumulation and expansion of repetitive sequences) in these proto sex chromosomes result in morphologically, as well genomically, specialized sex-specific chromosomes. It has also been proposed that sex chromosome formation may start with the acquisition of sexually antagonistic alleles close to the sex-determining locus which would suppress recombination and pseudogenize genes (i.e., multiply and accumulate these genes, with mutations that cause loss of functionality) that do not have sex-specific benefits [39]. Additional sexually antagonistic alleles could cause the expansion of the non-recombining region and further suppression of recombination [40,41,42] leading to an increase in the size of the Y or W. However, these increases are often reduced through large-scale deletions [43,44], resulting in a chromosome that is smaller than the X or Z chromosome [2,45]. 

Sex chromosomes are the most rapidly evolving structural entities of the genome in many groups of animals [12,34,36,46]. One aspect of sex chromosome evolution, recombination suppression, is known to trigger several evolutionary processes, such as Muller’s Ratchet [47,48] and genetic hitchhiking (see below in Section 2.1), that cascade through to the loss of gene activity and pseudogenization, particularly where sex chromosomes are heteromorphic. However, it is less clear what happens in the case of homomorphic sex chromosomes [39]. All sex chromosomes in the heterogametic systems (X, Y, Z and W) have differences in their evolutionary environments including background selection pressure, effective population sizes, mutation rates, and genomic imprinting as well as meiotic drive [45,46,47,48]. These factors influence chromosomal rearrangements, and thereby play a role in changes in genome structure [39,49]. For example, genes on the mammalian Y chromosome are subject to selection via expression in the male phenotype, whereas genes on the W chromosome in birds do not appear to be subject to the same level of selection primarily because Z chromosomes exhibit differential expression in males [49,50,51,52]. 

### 2.1. Hitchhiking, Meiotic Drive and Imprinting

Evolution of sex chromosomes and their ability to spread within populations have been explained using different mechanisms, such as genetic hitchhiking, meiotic drive and imprinting. However, none of these explain how initial processes such as the acquisition of sex-specific genes or the suppression of recombination occurs or how derived lineages end up in two genetically distinct sex chromosomes [53].

Genetic hitchhiking, in the context of sex chromosome evolution, is the process whereby genes or mutations in genes not involved in sex determination are carried along with the chromosome through linkage. In this process, deleterious mutations on the Y chromosome cannot be removed by recombination and are therefore able to spread through a population because they are linked to, and hitchhike with, sex-specific beneficial genes. The net result of this is that the heterogametic chromosome becomes less and less genetically active resulting in locus-specific dosage tolerance or compensation [3,54]. The homogametic chromosomes (X or Z) escape this fate because they are still able to recombine [43]. However, selective advantage can be gained by the heterogametic chromosome when beneficial mutations are more pronounced than the linked deleterious ones [43]. The hitchhiking effect is most distinct when Muller’s ratchet is in place, that is, when these mutations/changes are irreversible. 

Maintaining an even sex ratio among offspring is seen as being critical for many species, and it is common for the heterogametic sex to produce equal numbers of male and female gametes (X and Y or Z and W). Nevertheless, unequal transmission of X and Y chromosomes from individuals of the heterogametic sex have been observed during meiosis [55,56]. Such phenomena are referred to as sex chromosome ‘meiotic drive’ and result in biased sex ratios among offspring and even within populations [56]. Such phenomena may also occur in TSD species where skewed sex ratios can be caused by exposure to nest incubation temperatures that are biased towards one sex or the other [13,56]. However, the unequal distribution of sex chromosomes can reduce mean fitness within a population by interfering with the sex chromosome—autosome relationship (intragenomic conflict between the X, the Y chromosomes, and the autosomes) and altering the intensity or mode of sexual selection [56]. Nevertheless, such phenomena are neither evolutionary stable nor easily detectable, hence these might be a more common occurrence than reported (only to occur in the insect order Diptera and mammalian Rodentia) [57,58].

It has been proposed that differences between sexes may be determined by differential methylation in nuclear DNAs of males and females. Methylation suppresses recombination and increases mutation rates that drive Muller’s ratchet. As a result, selection pressures are created to remove these areas of Y or W chromosomes, ultimately playing a role in their degeneration [53]. Genomic imprinting is the epigenetic marks imprinted on genes owing to chromosomal transmission through the female and male germlines and this often results in gene expression differences between maternally and paternally inherited alleles [59]. These epigenetic marks are established during spermatogenesis and oogenesis by DNA methylation and histone modifications and carried from parents to offspring via sperm or egg cells. These are continued within the offspring somatic cells by mitosis [60]. As a result, gene expression in offspring depends on a parent-of-origin manner rather than from both homologous chromosomes—some genes are expressed that are only from the father and some others are expressed that imprinted only from the mother [61]. But a balanced contribution between these maternal and paternal expressions is required for development of such lineages [59].

### 2.2. Multiple Origin, Rapid Transitions and Turnovers

Sex determination in amniotes shows a sharp contrast between the extraordinary conservation of mammals and birds and the astonishing lability of reptiles [62]. Sex determination in therian mammals depends almost universally on the presence or absence of the Y chromosome and the master sex determining gene *SRY*, as demonstrated through manipulative studies of development and gene expression [63,64,65]. In marsupials too, the formation of the testes is determined by a Y-dominant mechanism, although other traits that are also characteristic of males and females depend on X-chromosome dosage [66]. Deterioration of the Y chromosome in mammals is ubiquitous leading to pronounced heteromorphy of the X and Y chromosomes or, in the case of mole voles (*Ellobius* sp.) and spiny rats (*Tokudaia* sp.) [67,68,69], the total loss of the Y. Birds too appear highly conserved in their sex determination mechanism. ZW sex chromosome heteromorphy occurs in most birds [70] and female development depends on the presence or absence of a W chromosome, since it carries female-determining genes. In contrast to mammals, male sex determination in chickens (sensu birds) have shown to be influenced by the dosage of the Z-linked gene *DMRT1* (two copies of the gene in males, one copy in females) [70] rather than through a dominance as in most eutherian mammals (Y-linked *SRY*, [63,65,71]). However, existence of ZZW females in birds (e.g., [72]) may indicate a more complex system, including a genic balance system, could not be ruled out. 

Against this background, sex determining modes among reptiles are diverse. Among those with GSD, both male and female heterogamety (XY and ZW) are known in turtles, female heterogamety (ZW, Z_1_Z_2_W, or ZW_1_W_2_) is known in snakes and both are known in lizards including XXY [73,74]. Many species have TSD where sex is determined by the temperature of incubation [75,76,77,78] and temperature and genotype can co-exist within species or even interact to reverse chromosomal sex [79,80,81,82] and influence sex ratios and drive divergence in sex determining mechanisms [82]. In contrast to mammals and birds, the sex chromosomes in most GSD reptiles are cryptic, lacking detectable heteromorphic chromosomes and in many cases involving micro-chromosomes. 

**Figure 1:**
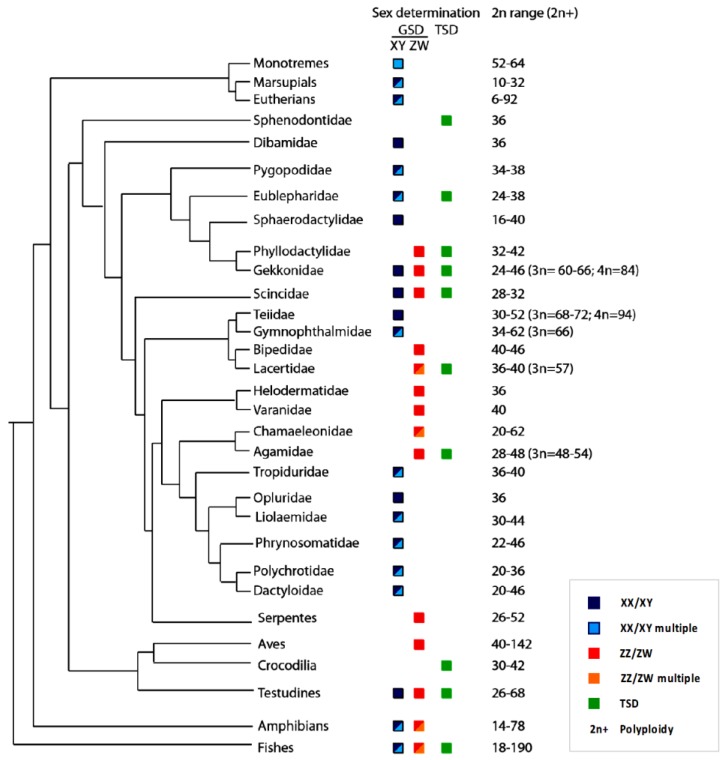
Truncated phylogeny (not according to scale) showing modes of sex determination and number of chromosomes in major lineages of vertebrates, with a particular emphasis on major families of lizards where modes of sex determination and sex chromosome systems are known and show high diversity. This figure includes only those lizard families where sex chromosomes have been identified cytogenetically. Adopted from [6,62,107,108,109,110,111,112,113,114,115,116,117] and references therein.

The diversity of sex determining mechanisms in the ectothermic reptiles, compared to that of the homeothermic birds and mammals, and amphibians and fish (typically poikilothermic), may have arisen because of a unique predisposition to the development of TSD, acting as an intermediary in the evolution of GSD in its various forms [83]. The diversity and haphazard distribution of sex determination mechanisms among reptiles and the lack of sex chromosome homology suggests that transitions between modes has occurred many times (Figure 1) [6,84] and may occur extremely rapidly [17]. The interaction between temperature seen in bearded dragons provides a mechanism for rapid switching among chromosomal states and TSD [17,19]. Among reptiles, lizards are perhaps the most diverse and labile. Of the 181 species for which sex chromosomes have been detected, about two thirds have male heterogamety [6], while both male and female heterogamety occur in at least one family (Gekkonidae) along with TSD [85]. This fascinating diversity of sex-determining mechanisms shows no clear phylogenetic segregation [13,14,86]. 

## 3. Overview of Current Understanding of Sex Determination and Sex Chromosomes in Lizards

### 3.1. Temperature-Dependent Sex Determination in Lizards

Unlike other amniotes, the class reptilia is the only vertebrate group where both GSD and TSD have been described (450 species examined) and where there is evidence of frequent evolutionary transitions between these two modes (Figure 1) [12,14,62]. Although not all species have been subjected to systematic incubation experiments to identify cases of TSD, it has been likely that all crocodiles (25 species), marine turtles (seven species), and tuatara (one species), and several freshwater turtles and tortoises (49 of 260 species) and many lizards (45 of 6459 species) have TSD. So far, TSD has not been reported in any snakes (3619 species), but only three species have been subjected to laboratory-based systematic incubation experiment [87,88,89,90], and at least one publication (though not focused on TSD) reported sex ratio biases in response to variable incubation temperatures in stripe-tailed rat snake *Elaphe taeniura* [91]. In that case, both high and low temperatures produced more males while intermediate temperatures produced females resulting in a biased sex ratio. TSD has also been described in both viviparous and ovoviviparous reptiles with three out of the four viviparous (lizards) and one ovoviviparous species (snake) that have been tested through incubation experiments reported to have TSD [87,88,92,93,94,95]. Most species remain untested for their sex determination system, highlighting the fact that what we know about reptilian TSD is only the tip of the iceberg. More lab-based systematic incubation experiments as well as field based studies will be required to obtain a clearer picture of reptilian TSD (for details see available literatures [5,12,13,18,21,22,36,38,45,62,84,86,96,97,98,99,100,101,102,103,104,105,106] and references therein).

### 3.2. Karyotypes, Genotypic Sex Determination and Sex Chromosomes in Lizards 

Reptilians are also karyotypically diverse, with macro- and micro-chromosomes occurring (with the exception of crocodilians which have all macrochromosomes) in the genomes of most reptile species that have been karyotyped to date. They are also karyotypically heterogeneous group [108] with varying numbers of micro- and macro-chromosomes (Table 1). The lowest number of diploid chromosomes occurs in a lizard (2*n* = 20, Cameroon stumptail chameleon, *Rampholeon spectrum*), while the highest diploid number (2*n* = 68) occurs in a freshwater turtle (twist-neck turtle, *Platemys platycephala*) and the highest number of macro- and micro-chromosomes are observed in crocodilians and freshwater turtles respectively (42 and 56 respectively, Table 1). Although diploidy is common in reptiles, a considerable number of parthenogenetic species have triploidy and the occurrence of triploid individuals in populations of diploid species is not uncommon [118]. 

Reptiles exhibit considerable genomic variation across different organizational levels of reptiles ranging from 1.03 to 5.3 Gb (Table 1) [119]. The lowest range of genome size is found in a lizard, in Mionecton skink *Chalcides mionecton* (1.03 Gb) and largest in Mediterranean spur-thighed tortoise *Testudo graeca* (5.3 Gb). The largest genome size in lizards is 3.80 Gb in Slow worm *Anguis fragilis.* However, consistence with different plant and animal groups [120], genome size and the number of micro and macro chromosomes are not evolutionarily correlated in reptiles. 

### 3.3. Sex Chromosome Differentiation in Lizards

In many lizards, as in most mammals and birds, the heterogametic Y or W chromosomes are highly differentiated morphologically and in sequence composition. In contrast, there is great variability in the degree of differentiation between the sex chromosome homologues in reptiles, particularly in lizards [121]. In addition, some lizards have been found to possess complex male or female heterogametic systems involving multiple sex chromosomes from varying evolutionary stages of differentiation [84]. In particular, heterochromatinization of one sex chromosome varies greatly in GSD lizards, ranging from a small block to the entire chromosome. Additionally, chromosomal rearrangements such as fusions, inversions and translocations have also contributed to sex chromosomal differentiation in lizards [122]. 

Heteromorphic sex chromosomes are differentiated at the level that can usually be detected cytologically, while in the case of homomorphic sex chromosomes, this differentiation is most likely at the gene level. It has been found that the W chromosome of *P. vitticeps* is heterochromatic and more heavily C-banded than its Z chromosome. This heterochromatinization is thought to be an early change that initiated sex chromosomal differentiation in lizards [121]. On the other hand, deletion events are likely to be involved in the differentiation of W chromosomes in multiple agamid lizard species including *P. vitticeps, Pogona barbata, Amphibolurus nobbi* and *Ctenophorus fordi* [84].

It is generally considered that highly differentiated sex chromosomes are a barrier to the subsequent evolution of TSD, and that homomorphic sex chromosomes are a necessary prerequisite for such a transition in a sex-determining mechanism [4,36,123]. However, GSD reptiles with highly differentiated sex chromosomes can indeed switch to TSD as in the case of *P. vitticeps* and *Bassiana duperreyi* [121,124]. Moreover, Chromosome rearrangements may well play a major role in sex chromosome differentiation in the reptilian lineages [121]. Shifts have even been observed from one form of agamid lizard ZW sex chromosome system to a different ZW system in a short evolutionary time [84]. 

## 4. Unique Pathway of Sex Chromosome Evolution in Lizards—A Different Pathway from the Classical Model (as That Proposed for Birds and Mammals)?

### 4.1. Temperature Dependent Sex Determination and Sex Chromosome Evolution

Sex in TSD species is determined by temperature experienced by developing embryos and any involvement or association of sex chromosome is unknown [4,13,36]. It is predicted that the common ancestral mechanism for all amniotes was GSD with ZW heterogamy, although the TSD found in present day squamates have been known to evolve multiple times and independently from a common TSD ancestor [13,14]. However, it seems unlikely that sex in any species is determined purely by TSD and transitions between TSD and GSD may involve loss and gain of sex chromosomes as described in Section 2. Transitions between these two mechanisms are more likely to occur in species with poorly differentiated sex as in some reptiles and it is also likely that well developed sex chromosomes will resist such transitions owing to their accumulation of beneficial sexually antagonistic genes that maintain and regulate sexual fitness, meiosis and dosage compensation [111].

However, in reptile species with well-differentiated heterogametic sex chromosomes, as in Australian bearded dragon *P. vitticeps*, sex reversal has been observed both in the laboratory and the field, and has been attributed to the influence of temperature during embryonic development [17,19]. Normal ZZ males incubated at high temperatures became sex-reversed to fertile ZZ females that are able to breed with ZZ males. The homomorphic sex chromosomes in ZZ male and female offspring have effectively become autosomal [17]. This finding shows how temperature could cause a rapid transition from GSD to TSD and in doing so, eliminate the W chromosome. A similar case was observed in the Eastern three-lined skink, *B. duperreyi*, with a well-differentiated XX/ XY GSD system where sex reversed XX were found in cold conditions [16]. In both lizard species, sex reversal took place in homogametic sexes and there was no sex reversal in the heterogametic sexes apparently. This prevents mating between individuals of heterogametic genotype and ultimately eliminates the chances of producing nonviable WW or YY offspring [81]. How this works in nature remains problematic and is in need of intense field-based studies to unravel.

### 4.2. Multiple Sex Chromosomes in Lizards

The existence of non-homologous sex chromosomes in closely related lineages implies that sex chromosomes in lizards can evolve independently and will be little constrained by past evolutionary events [125,126]. As a result, deviation from the typical XY/XX or a ZW/ZZ systems that includes absence of a sex chromosome from the system (X0/XX or Z0/ZZ systems) or multiple sex chromosomes (Z_1_Z_2_W/Z_1_Z_1_Z_2_Z_2_ and XY_1_Y_2_/XX systems) are possible. The number of chromosomes is considered as an important feature of eukaryote genomes which may have potential consequences for processes such as recombination and segregation [49]. Chromosome number may vary between closely related species and even within species that can contribute to adaptation and speciation [1,2,3,4,5,49]. Differences in chromosome numbers are usually caused by reciprocal translocation between two chromosomes—by the fusion between two acrocentric chromosomes or the split (fission) of a metacentric chromosome into two [127]. Fixation of chromosomal rearrangements through random genetic drift, changes in recombination rate and meiotic drive are the evolutionary forces that may be involved in such chromosome number variations [128,129] however, what allows fusion and fission to become fixed within a population is not yet known [49].

The fusion of a Y chromosome with an autosome creates an X_1_X_2_Y system with the unfused homologue segregating as a neo-X chromosome and causes an odd number of chromosomes in one sex [2,18]. Instances of such fusions have been found in a number of lizard families Gekkonidae (e.g., ♂33♀34 in *Phyllodactylus lanei*), Gymnopthalmidae (e.g., ♂57♀58 in *Calyptommatus* spp.), Chamaeleonidae (e.g., ♂34♀35 in *Bradypodion ventrale*), Iguanidae (e.g., ♂31♀32 in *Sceloporus* spp.) and Pygopodidae (e.g., ♂33♀34 in *Lialis burtonis*). Likewise, X-autosome fusions generate XY_1_Y_2_ systems, as may be in family Scincidae (e.g., ♂31♀30 in *Mabuya mabouya*), W-autosome fusions generate Z_1_Z_2_W systems as in Lacertidae (e.g., ♂36♀35 in few populations of *Lacerta vivipara*) and Z-autosome fusions generate ZW_1_W_2_ systems that have not yet been observed in lizards but probably occur in another reptile group, sea snake family Hydrophiidae (e.g., ♂34♀35 in *Hydrophis fasciatus*) [108]. Many species with sex chromosome-autosome fusions have been discovered as these multiple sex-chromosome systems can be easily identified [2,19,20,21,22]. Such phenomena can predominantly be found in reptiles among amniotes and especially among lizards. 

### 4.3. Sex Chromosome Evolution in Lizards May Involve yet Undescribed Gene Regulatory Mechanisms

The evolution of heteromorphic sex chromosomes in vertebrates has been thought to involve a process of gradual degradation of macro-chromosomes [2,3,11,31,35,38]. However, both macro- and micro-chromosomes have been identified as sex chromosomes in reptiles [121,130,131,132,133,134,135,136]. In addition, multiple sex chromosomes are common in some groups, particularly in Iguanids and Lacertids, suggesting that the translocation of sex chromosomes to autosomes has occurred. These variations present a challenge for interpretation under the conventional theory of vertebrate sex chromosome evolution, while morphological variations between sex chromosomes also range quite substantially from homomorphic to highly heteromorphic, representing various stages of evolutionary degradation [8,11,121]. However, this does not often correlate with the evolutionary age of those taxa. The identification of sex micro-chromosomes has previously been challenging but is now made possible owing to recent advancements of cytogenetic techniques (such as CGH—Comparative Genomic Hybridization and FISH—Fluorescence in situ Hybridization). Whether similar mechanisms are involved in the evolution of micro sex chromosomes has yet to be discussed. Therefore, molecular and cytogenetic mechanisms behind the evolution of sex micro-chromosomes remain unknown, such as whether they have evolved as a result of chromosome fission or fusion events or micro-chromosomes also followed the same pathway as proposed for vertebrates, or whether completely different molecular mechanisms are involved in evolution of sex microchromosomes, is yet to be determined. One possibility could be that both macro and microchromosomes have evolved independently, involving new pairs of sex microchromosomes driven by frequent transitions, either via translocation of sex determining factors via transposition or novel sex chromosomes have evolved after each transitions, involving novel genes and novel chromosomes.

**Figure 2:**
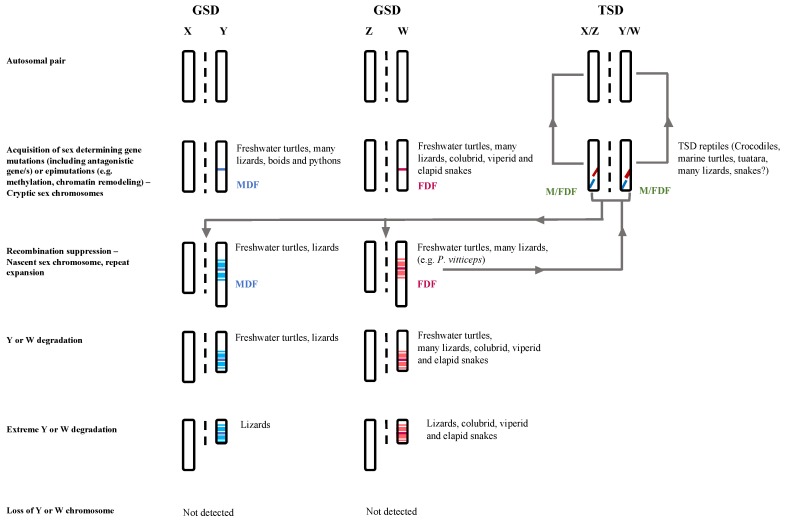
Proposed model for lizard sex chromosome evolution, modified from [11]. Many Reptiles are likely to have followed the currently held view of sex chromosome evolution as proposed for vertebrates but may also involve other regulatory molecular mechanisms (e.g., epigenetic). The sex determination (sensu sex chromosomes) in genotypic sex determination (GSD) and temperature-dependent sex determination (TSD) with environment influence is bipotential and could involve polygenic or epigenetic mechanisms, hence retaining the homomorphic sex chromosomes and high diversities. MDF = Male determining factor, FDF = Female determining factor.

We argue that, in addition to the proposed pathway of vertebrate sex chromosome evolution, the evolution of sex chromosomes in reptiles has also occurred via other molecular mechanisms, particularly subtle gene regulatory mechanisms (e.g., epimutations, i.e., abnormal transcriptional repression of active genes and/or abnormal activation of usually repressed genes caused by errors in epigenetic gene repression [137], evolution of sexually antagonistic genes, e.g., [53,138]). This perhaps suggests reptiles possess a plasticity in maintaining sex ratios in highly labile environments (Figure 2), which would somewhat explain the maintenance of TSD, transitions between TSD and GSD as well as temperature mediated sex reversal in GSD species with cryptic (*P. vitticeps*) and heteromorphic sex chromosomes (*B. duperreyi*). This also explains the numbers of homomorphic sex chromosomes (in about 70 spp.) as well undetected sex chromosomes (1185 spp.) in described karyotypes (1562 spp.) [139].

## 5. Conclusions

Reptiles have diverse modes of reproduction, sex determination mechanisms as well as diversity of sex chromosomes—from GSD to TSD as well as GSD with temperature influences. Independent evolution and multiple lineage divergence in reptiles than other amniotes (mammals and birds) may have contributed in the diversified systems ranging from species with devoted sex chromosomes (homomorphic, XY/ZW heteromorphic or multiple sex chromosomes) to none, that is, non-strong genetic determinant, autosomal genes acting differently on sex-determining pathways. However, it has been observed that sex chromosomes have degenerated and novel sex chromosomes have evolved to resolve sex determination. There are also numerous instances of convergent evolution of sex chromosomes across distantly related taxa if certain genes are particularly adept at taking on a sex-determining role [140,141].

Several species of reptiles have been studied in respect to evolution of sex chromosomes but unlike other amniotes as birds and mammals, reptiles still lack a functional model that represents the overall reptilia. Species with well-characterized GSD, such as green anole or *P. vitticeps* should be given priorities in identifying master sex genes in reptiles, as TSD in reptiles might be polygenic involving multiple sex chromosomes or even autosomes [142]. Reptilian sex chromosomes have similar evolutionary history but have taken different pathways with differential temperature influences, and particularly in lizards.

Recent advanced technologies such as comparative gene mapping and whole genome sequencing have shown surprising relationships among different groups of reptiles, as well as with other amniotes that share common ancestry. Therefore, it will be valuable to compare maps and sequences and explore homologies across the phylogeny. In addition, the traditional approaches such as incubation experiments and chromosome analysis by karyotyping sets the bases of chromosomal studies and are no less important. Furthermore, other recent technologies such as transcriptomics and methylomics that measure gene expressions, chromosomal modifications and epigenetic expressions may provide the further integration necessary to increase our understanding of complex sex chromosome evolution in lizards and reptiles as a whole.

## Figures and Tables

**Table 1:** Ranges of diploid chromosomes numbers and numbers of macro and microchromosomes and genome sizes in major groups of reptiles.

Taxon	Chromosome			Genome Size (Gb) [119]	
	2*n* Range	Macro Range	Micro Range	Low	High
Tuatara	36	28	8	4.9	
Lizards	20–62	10–38	0–28	1.03	3.8
Snakes	26–50	10–38	0–36	1.3	3.7
Crocodilians	30–42	30–42	0	1.3	3.9
Freshwater Turtles	26–68	10–36	0–56	1.4	5.3
Marine turtles	56	24–32	24–32	2.6	
